# Composition Formulas of Cu-Ni-Al Cupronickel Alloys Derived by the Cluster Plus Glue Atoms Model

**DOI:** 10.3390/ma18184288

**Published:** 2025-09-12

**Authors:** Xiaolin Cheng, Mengxin Zhao, Yajun Zhao, Chuang Dong

**Affiliations:** 1 School of Materials Science and Engineering, Dalian Jiaotong University, Dalian 116028, China; chengxiaolin2021@163.com (X.C.); 19741163451@163.com (M.Z.); dong@djtu.edu.cn (C.D.); 2 School of Materials Science and Engineering, Dalian University of Technology, Dalian 116024, China

**Keywords:** Cu-Ni-Al cupronickel alloys, cluster plus glue atoms model, cluster formula, volume fractions, Young’s modulus

## Abstract

Cu-Ni-Al cupronickel alloy is a precipitation-strengthening alloy with γ′ (Ni_3_Al) and γ″ (Ni_3_Nb) phases embedded in the γ phase (Cu solid solution), enhancing strength and corrosion resistance. However, it is difficult to clarify the correlation between composition and properties due to the complex elements and microstructure. This study attempts to construct the composition formulas and microstructure constitution (e.g., phase volume fractions) of Cu-Ni-Al cupronickel alloys via the cluster plus glue atoms model. Based on the solubility behavior of alloying elements, a systematic classification was established as follows: γ phase elements (Cu-like elements, including Cu, Ni, Al, Mg, Mn); γ′ phase elements: Al-like elements (including Al, Nb, Si, Cr, Mn, Fe); and Ni-like elements (including Ni, Fe, Cu). Using this classification and phase composition (γ and γ′ phase under heat-treated conditions), the cluster formula of the structural units that carry the composition information were formulated as follows: γ-{(Cu,Ni,Al,Mn,Mg)_16_}_1-x_ and (γ′ + γ″)-{(Al,Nb,Si,Cr,Mn,Fe)_4_(Ni,Fe)_8_Cu_4_}_x_, where x represents the volume fraction of precipitates (γ′ + γ″). The representative Cu-Ni-Al cupronickel alloys were also analyzed by the cluster formula, and theoretical phase volume fractions were obtained (13.21–26.32%). Furthermore, Young’s modulus, predicted by the cluster formula, shows good agreement with the practical alloys, verifying its applicability for alloy design.

## 1. Introduction

Cu-Ni-Al cupronickel alloys are widely used as structural materials, such as tank gears [1] and ship bolts [2], serving in extreme temperature environments (−196 °C to 300 °C) and/or under complex stress conditions [3], due to their excellent corrosion resistance, high strength, and toughness, as well as good wear and hydrogen embrittlement resistance. The Columbia Company [4] developed the first industrial Cu-Ni-Al cupronickel alloy (Cu-15Ni-3Al) in the 1940s, leading to successful applications in submarines and pipelines. To date, six representative grades have been developed (see Table 1); their compositions can be summarized as primary alloying elements ranging from Cu–(5.5–25) Ni–(1.0–3.0) Al, with the addition of Fe, Mn, Nb, Si, Cr, and Mg. The microstructure is characterized by the coherent precipitation of the γ′ phase (Ni_3_Al, Pm-3m) [5] within the γ phase (Cu solid solution, Fm-3m), providing excellent mechanical properties (tensile strength: 600–900 MPa; elastic modulus:130–155 GPa; elongation: 8–18%). Considering the fast evolution of industrial alloys, composition and performance data are indeed subjected to frequent updates. The relevant data for Alloys 1 to 6 in Table 1 are taken from up-to-date sources as follows:

Alloy 1 and Alloy 2: BAl6-1.5, BAl13-3 are consistent with those reported in the 2025 Russian “https://evek.biz/materials/splav-mna13-3-kunial-a.html (accessed on 8 September 2025)”.

Alloy 3: UNS C72400-Hiduron 130 aligns with information from Langley Alloys in 2025 “https://www.langleyalloys.com/products/hiduron-130/ (accessed on 8 September 2025)”.

Alloy 4: UNS C72400 is consistent with the 2022 “https://store.astm.org/b0422_b0422m-22.html (accessed on 8 September 2025)”.

Alloy 5: UNS C72420-Hiduron 191 matches the latest 2025 company standard “https://www.langleyalloys.com/products/hiduron-191/ (accessed on 8 September 2025)”.

Alloy 6: UNS C72420-Marinel 220 agrees with the composition and properties reported in 2025 by BNM Ltd(England, South Yorkshire) “https://www.tachart.com/material/marinel-220/ (accessed on 8 September 2025)”.

The development of the Cu-Ni-Al cupronickel alloys primarily focused on modifying the content and ratio of γ′ phase forming elements, Ni and Al. Based on Cu-15Ni-3Al, the high strength and toughness alloy UNS C72400 was developed by the American Society for Metals [6,7], with a composition standard of 11.0–15.0 wt.% Ni and 1.5–2.5 wt.% Al. The derivative alloy UNS C72400-Hiduron130 [8] further improved the composition range to 13.0–16.0 wt.% Ni and 2.3–3.0 wt.% Al. Subsequently, Clark et al. [2] developed the UNS C72420 (Hiduron 191), achieving enhanced elongation by reducing the Al content to 1.0–2.0 wt.%, while increasing Mn to 4–5 wt.% and Fe to 0.8–1.5 wt.%, respectively. In 1996, Grylls et al. [9] increased the Ni content to 18–25 wt.% while maintaining the Al at 1.0–2.0 wt.%, developing the derivative alloy of UNS C72420-Marinel 220. Similarly, the BAl grade has also been developed in the Chinese alloys system (designated as MNA in Russian), including: BAl13-3 (MNA13-3) [10,11], with Al content reduced to 2.3–3.0 wt.% and a minimum Ni content of 13.5 wt.%, providing a high tensile strength of 900 MPa; and BAl6-1.5 (MNA6-1.5) [10,11], with Al content ranging from 1.2 to 1.8 wt.% and 5.5–6.5 wt.%Ni, exhibiting high strength (630 MPa) and excellent plasticity (elongation: 8%).

**Figure 1:**
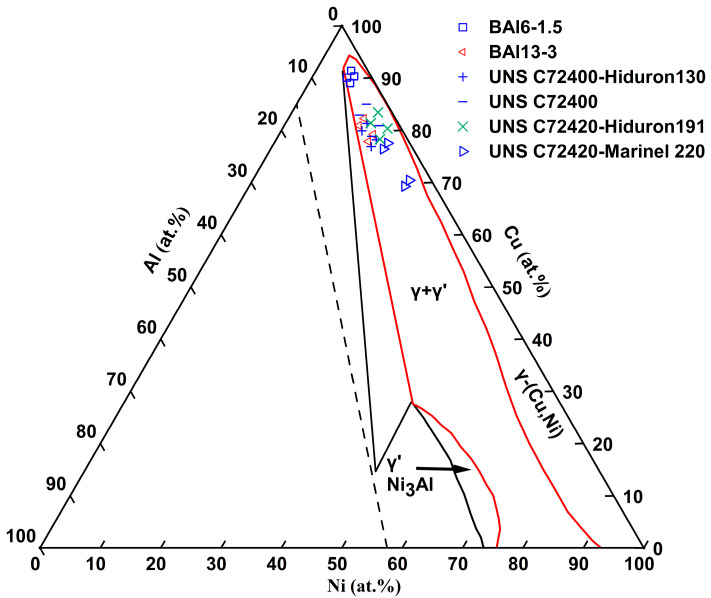
Room-temperature Ni-Cu-Al ternary phase diagram [12], highlighting the composition range of major alloying elements (Ni and Al) in Cu-Ni-Al cupronickel alloys within the dual-phase (γ + γ′) region outlined in red.

**Figure 2:**
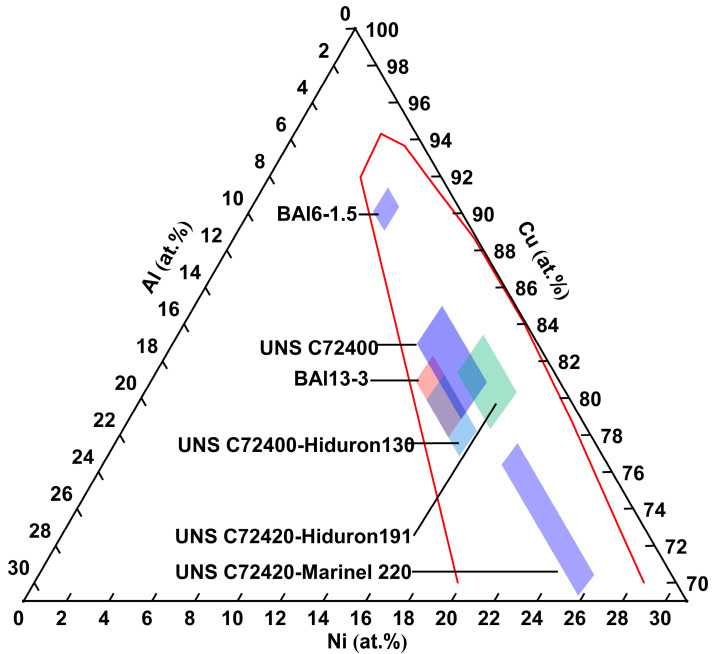
Enlarged Ni-Cu-Al ternary phase diagram with composition zone (Ni and Al) of Cu-Ni-Al cupronickel alloys.

The composition standards and mechanical properties of Cu-Ni-Al cupronickel alloys are also summarized in Table 1. The Ni content varies between 5.7 and 26.2 at.% (5.5–25.0 wt.%), while the Al content ranges from 2.3 to 6.8 at.% (1.0–3.0 wt.%). The composition range of Cu-Ni-Al cupronickel alloys for the major elements Ni and Al is shown in the room-temperature Ni-Cu-Al ternary phase diagram [12] (see Figure 1). These representative industrial alloys are composed of γ and γ′-Ni_3_Al phases. Figure 2 shows an enlarged view of the Ni and Al composition range. Comparing UNS C72400-Hiduron 130 and UNS C72420 (Hiduron 191), the Ni content is 13.5–16.4 at.% (13.0–16.0 wt.%), and the Al content of the latter decreases from 5.2 to 6.7 at.% (2.3–3.0 wt.%) to 2.3–4.5 at.% (1.0–2.0 wt.%), resulting in a rapid decrease in tensile strength and increase in elongation. A similar trend is observed in BAl6-1.5 and UNS C72420 (Hiduron 191), which have the same Al content range of 2.3–4.6 at.% (1.0–2.0 wt.%). When the Ni content increases from 5.8 to 6.8 at.% (5.5–6.5 wt.%) to 14.3–17.2 at.% (13.5–16.5 wt.%), the elongation increases (from 7% to 18%), accompanied by a rise in Young’s modulus.

Noting the above, both weight percent (wt.%) and atomic percent (at.%) were employed in the present paper. Weight percent (wt.%) was used when discussing industrial standards and productions, as summarized in Table 1. Alloy specifications and production parameters are universally defined in wt.%, making it the most practical form for comparing commercial alloys and ensuring practical applicability. Meanwhile, atomic percent (at.%) was employed in the present study for composition study and cluster formula construction. In the present work, the composition formulas and microstructure constitution of Cu-Ni-Al cupronickel alloys were constructed via the cluster plus glue atoms model, which provides the interpretation of the alloy structure from the atomic level. For this purpose, weight percent composition (wt.%) for industrial alloys was converted into atomic percent (at.%) to clarify the study on the atomic scale.

**Figure 3:**
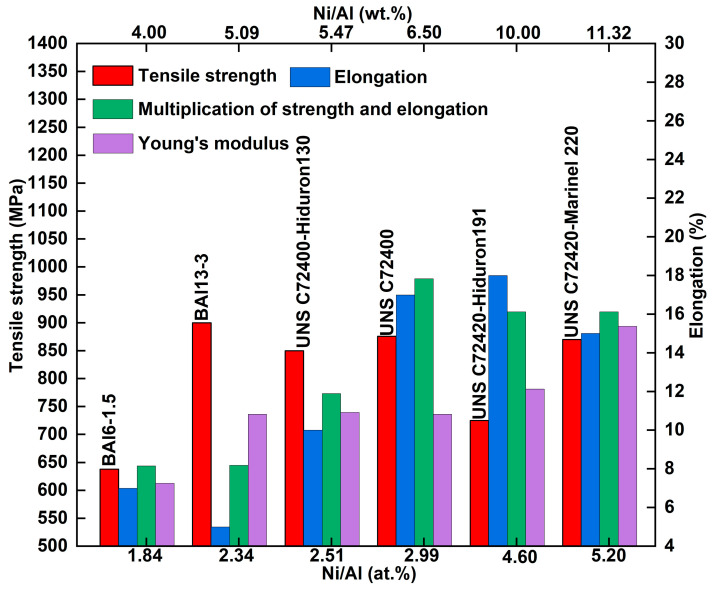
Relationship between Ni/Al atomic ratio and mechanical properties of Cu-Ni-Al cupronickel alloys.

As previously mentioned, the typical microstructure of Cu-Ni-Al cupronickel alloys is the coherent precipitation γ′ phase (Ni_3_Al) within the γ phase. Therefore, the content and ratio of γ′ phase forming elements (Ni and Al) regulate the properties. The trend between the mechanical properties and the Ni/Al ratio of Cu-Ni-Al cupronickel alloys is summarized in Figure 3. In 1933, Griffiths et al. [13] first investigated the relationship between the Ni/Al weight ratio and hardness. Subsequently, Clark et al. [2] found that the maximum strength was achieved at a Ni/Al weight ratio of 2.30 (atomic ratio 5.0). Ferguson et al. [14] indicated that increasing the Ni/Al atomic ratio could enhance the thermal stability and hardness, leading to discontinuous precipitation at the grain boundary and a transition from adhesive to abrasive wear. Recently, Dong et al. [15] designed the Cu-15Ni-(1–4) Al alloys to adjust the Ni/Al ratio; their study concluded that at a Ni/Al weight ratio of 1.72 (atomic ratio 3.75), discontinuous precipitation will emerge and disrupt the grain boundary migration and continuity. Tuck et al. [16] proposed that the complete formation of the γ′-Ni_3_Al phases occurs at a Ni/Al weight ratio of 6.52 (atomic ratio 3). As the Ni/Al weight ratio increases further, excessive Ni atoms dissolve into the γ phase (Cu solid solution). When the Ni/Al weight ratio ranges from 8.5 to 9.5 (atomic ratio 3.91–4.37), the γ and γ′ phases yield the best mechanical properties. Zhang et al. [17] adjusted the Ni/Al atomic ratio from 1 to 3 while fixing 80 at.% Cu, finding that the maximum result (multiplying electrical conductivity by tensile strength) occurred at a Ni/Al atomic ratio of 2 (weight ratio 4.36). As shown in Figure 3, industrial alloys exhibit Ni/Al weight ratios of 4.0–11.32 (atomic ratio 1.84–5.20), with the highest multiplication of strength and elongation (green rectangle in Figure 3) achieved at a Ni/Al weight ratio of 6.52 (atomic ratio 3).

In addition to the γ′ phase forming elements (Ni and Al), alloying additions such as Nb, Fe, Cr, Mn, and Si are conventionally categorized into two groups: precipitation-strengthening elements (forming Ni_3_Al and Ni_3_Nb) and solid solution-strengthening elements (dissolved in the Cu matrix). Due to the complex partitioning of these elements between the γ and γ′ phases, it is impossible to determine the precipitates’ volume fraction directly from alloy composition. Furthermore, establishing a direct correlation between precipitate volume fraction and mechanical properties is particularly challenging due to the ultrafine γ′ precipitates (20–50 nm in diameter). Previous studies, such as Christofidou et al. [8], reported the precipitates’ volume fraction range as ~10–50% for Cu-Ni-Al cupronickel alloys, but lacked precise quantification. This study attempts to define the structural units and the precipitates’ volume fraction of Cu-Ni-Al cupronickel alloys using the cluster plus glue atoms model, including BAl6-1.5, BAl13-3, UNS C72400, Hiduron130 (C72400 derivative), UNS C72420, and Marinel 220 (C72420 derivative). Finally, a cluster-formula-based method is developed to correlate composition, phase fraction, and Young’s modulus. In industrial production, screening for an alloy with comprehensively excellent properties involves enormous costs. Moreover, the establishment of composition standards (wt.%), which are crucial for controlling alloy properties, often relies heavily on extensive experimental results and lacks efficient and straightforward theoretical frameworks for understanding such standards (wt.%). By categorizing alloying elements and regulating the content of precipitate phases, the proposed cluster approach offers a novel strategy for understanding composition standards and optimizing alloying element design for industrial production. The present model constructs the correlation of composition and properties for industrial alloys from the physical basis, which will provide guidance for the high-efficiency design of new alloys, greatly reducing the production costs.

## 2. Ideal Cluster Formula of Cu-Ni-Al Ternary Alloys

### 2.1. Theoretical Composition Formula Based on the Cluster Model

**Figure 4:**
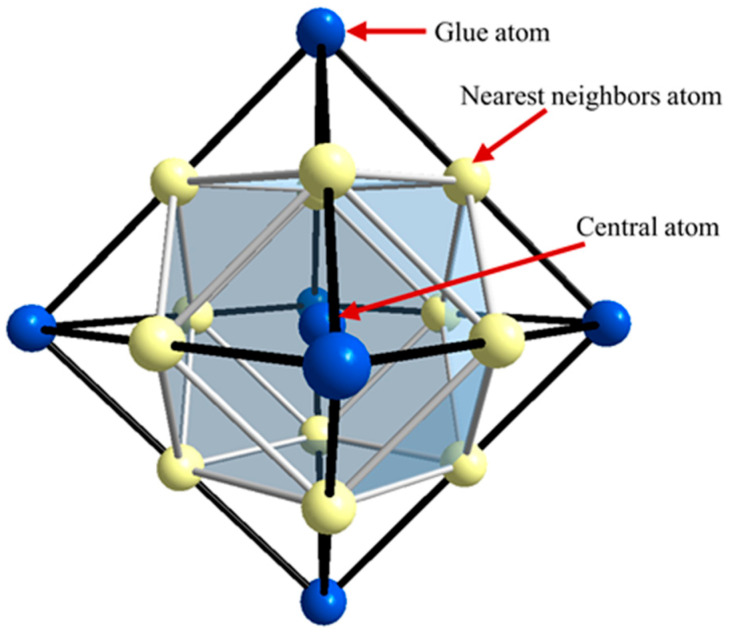
Cluster structure unit of FCC solid solution: center atom, the nearest cluster (shaded cuboctahedron; yellow spheres), and the glue atoms at the next-neighbor sites (octahedron; blue spheres).

Industrial alloys are mostly based on solid solutions characterized by chemical short-range ordering (SRO), which carries the structural information including crystal structure, chemical composition, and electronic structure. Based on the Friedel oscillations [18], Dong [19] proposed that SRO structures can be described by the molecule-like structural units confined to nearest-neighbor coordination polyhedral clusters, supplemented by a limited number of next-nearest-neighbor glue atoms. This is formalized through the cluster formula [cluster] (glue atoms)_x_, where x denotes glue atom. Specifically for FCC solid solutions, the cluster formula comprises 16 atoms: a center atom surrounded by the CN12 cuboctahedron, with 3 glue atoms occupying six next-neighbor sites, as illustrated in Figure 4. Noting that glue atoms are shared by neighbor clusters, half of them were assigned to each cluster. Recently, the cluster plus glue atoms model has been successfully applied to cupronickel alloys (C71500) [20], Ni-based superalloys (Inconel 718) [21], and dual-phase Ti alloys (Ti-6Al-4V) [22].

**Figure 5:**
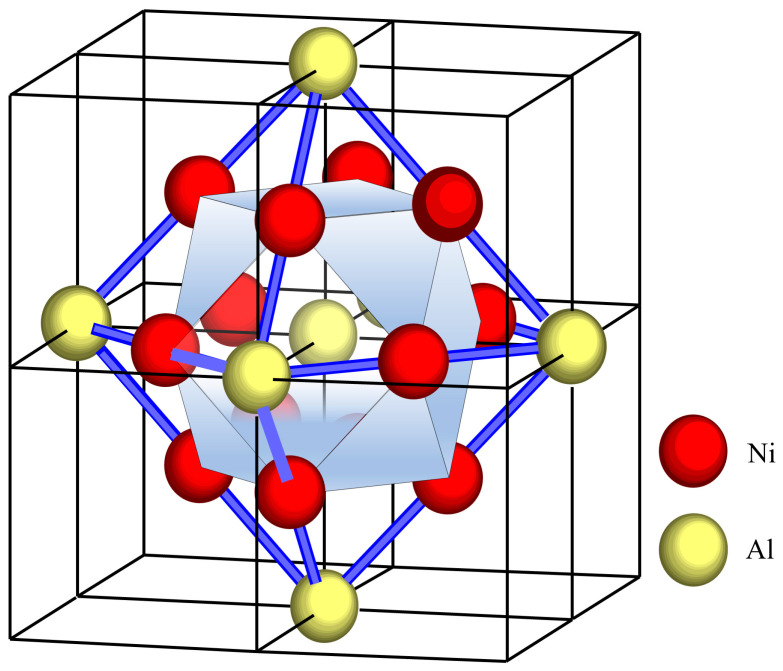
Theoretical cluster unit of γ′-Ni_3_Al solid solution: center atom (Al), 12 Ni atoms (red spheres) as the nearest cluster (cuboctahedron), and the 6 glue atoms (Al) at the face center location; the effective number of glue atoms for the cluster unit is 3 (i.e., 6 × 1/2), because they are shared by neighbor clusters; the cluster formula is expressed as [Al-Ni_12_] Al_3_.

The typical microstructure of Cu-Ni-Al alloys consists of the γ phase (Cu) and the γ′ phase (Ni_3_Al). In the cluster structure unit, atomic occupancy at the center, shell, and glue atom sites is determined by the enthalpy of mixing (∆H) and the relative content between the solute and solvent atoms [19]. For the γ′ phase (Ni_3_Al), the solute (Al) and solvent (Ni) atoms show a strongly negative ∆H (ΔH_Ni-Al_ = −22 KJ/mol [23]), leading to strong attractive interactions. In the theoretical cluster structural unit, 1 Al atom occupies the center atom site, 12 Ni atoms populate the nearest-neighbor shell, 3 Al atoms occupy the next-neighbor sites (Figure 5), and the cluster formula is [Al-Ni_12_]Al_3_ (simplified as {Al_4_Ni_12_}). The theoretical cluster structural units for the γ phase of Cu-Ni-Al alloys resemble the FCC solid solution (Figure 4). The major solute atoms in the γ phase are Ni and Al, and their contents are determined by the atomic ratio (Ni/Al). When the atomic ratio (Ni/Al) deviates from 3:1, the γ phase transitions into either Cu-Ni or Cu-Al solid solutions. Correspondingly, the general cluster formula of the γ phase is γ-{(Cu,Ni,Al)_16_}.

### 2.2. Composition Interpretation of Real Cu-Ni-Al Ternary Alloys

The term “real Cu-Ni-Al ternary alloys” was employed to describe the real Cu-Ni-Al alloys, not the theoretical Cu-Ni-Al ternary composition system. The theoretical cluster formulas of the γ and γ′ phases are formulated as follows: γ-{(Cu,Ni,Al)_16_}and γ′-{Al_4_Ni_12_}. Thus, the 16-atom cluster formula for Cu-Ni-Al alloys is expressed as γ-{(Cu,Ni,Al)_16_}_1-x_ + γ′-{Al_4_Ni_12_}_x_, where x represents the volume fraction of γ′ phase cluster units. In the alloy composition (100 at.%), x equals the sum of Al and Ni contents (at.%) in the γ′ phase. Since the phase composition (determining the cluster units) is influenced by fabrication processes such as heat treatment, this leads to significant variations in the partitioning ratios of alloying elements between the γ and γ′ phases. Therefore, the theoretical cluster model requires correction based on the actual phase composition near the final aging temperature of 500 °C for Cu-Ni-Al cupronickel alloys (Table 1).

As shown in Table 1, the industrial alloys commonly undergo aging treatment at 500 °C, and the obtained microstructures are employed as the service state. Industrial alloys can serve stably for over 10 years in extreme environments ranging from −196 °C to 300 °C with stable performance, verifying that after heat treatment at 500 °C, the γ′ phase has been fully formed, and the alloying elements are essentially stable, so the effect of temperature on the redistribution between phases is negligible. In the present study, the interpretation of Cu-Ni-Al alloys was conducted corresponding to the compositional–microstructural state of these alloys at 500 °C.

**Figure 6:**
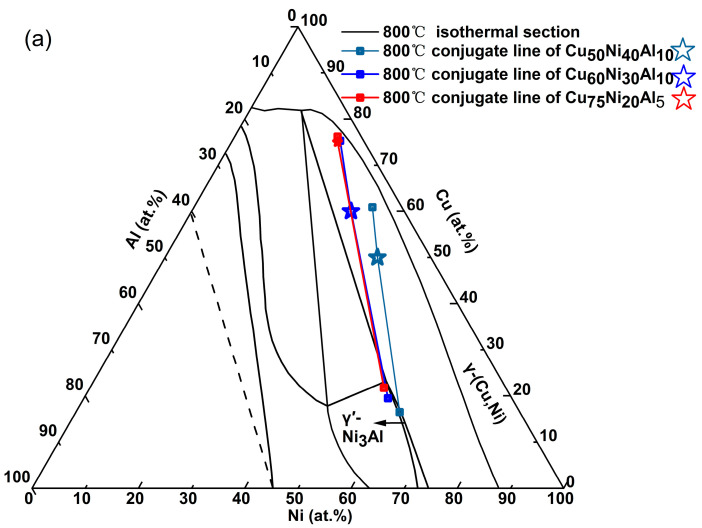

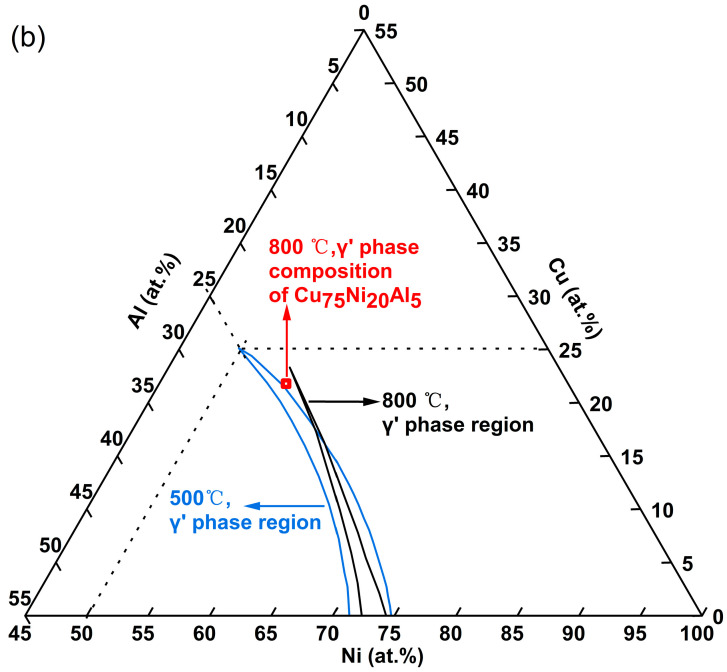
(**a**). Experimental compositions [24] of γ and γ′ phases plotted with conjugate lines for three alloys (Cu_75_Ni_20_Al_5_: red rectangle; Cu_50_Ni_40_Al_10_: light blue star; Cu_60_Ni_30_Al_10_: navy blue star) in the Ni-Cu-Al ternary phase diagram [12] at 800 °C. (**b**) Enlarged view of the γ′ phase region, comparing phase boundaries at 800 °C (black) and 500 °C (blue), with the composition of the γ′ phase of Cu_75_Ni_20_Al_5_ (red rectangle) at 800 °C.

Wang [24] measured the compositions of the γ and γ′ phases in Cu_50_Ni_40_Al_10_, Cu_60_Ni_30_Al_10_, and Cu_75_Ni_20_Al_5_ alloys at 800 °C (measurement error ≤ 3 at.%). The data are plotted on the 800 °C Ni-Cu-Al ternary phase diagram [12] (Figure 6a), showing that the γ and γ′ phase compositions lie on opposite sides of the alloy composition along the same conjugate line. The γ′ phase contains ~20 at.% Cu, while minimal Al and Ni dissolve in the γ phase. This confirms that Cu must be included in the actual cluster formula of the γ′ phase. Notably, the conjugate lines for Cu_60_Ni_30_Al_10_ and Cu_75_Ni_20_Al_5_ nearly coincide, with their γ′ phase compositions being highly similar. Recent work by Zhang [17] on Cu-Ni-Al alloys with 80 at.% Cu (aged at 500 °C) further revealed that elemental variations in the γ′ phase are confined to ≤10 at.%. These results collectively indicate that when Cu content exceeds 60 at.%, the γ′ phase composition stabilizes within a narrow range.

For high-Cu alloys (Cu > 60 at.%), such as Cu_75_Ni_20_Al_5_, the γ′ phase composition aligns with the tip of the 800 °C γ′ phase field (red rectangle in Figure 6a). Figure 6b provides an enlarged view of this region, overlain with the 500 °C γ′ phase boundary (blue line). Upon cooling from 800 °C to 500 °C, the γ′ phase field expands toward higher Cu concentrations, with its tip shifting to Cu_25_Ni_50_Al_25_ (intersection of dotted lines in Figure 6b). Based on this behavior, the 16-atom cluster formula of the γ′ phase (500 °C) is revised to [Al-Ni_8_Cu_4_]Al_3_ (simplified as {Al_4_Ni_8_Cu_4_}), consistent with the experimentally observed γ′ phase composition (Cu_25_Ni_50_Al_25_) in high-Cu alloys (Cu > 60 at.%).

It also indicates that a maximum of 25 at.% Cu can exist in the γ′ phase, meaning the 16-atom cluster formula contains up to 4 Cu atoms. The Al:Ni:Cu ratio in the γ′ phase (500 °C) is 1:2:1. If the Ni/Al atomic ratio is 2 (indicating complete γ′ phase formation by Ni and Al), the γ′ phase cluster units reach the maximum content. **When the Ni/Al atomic ratio ≥ 2**, excess Ni dissolves into the γ phase, and the volume fraction x of γ′ phase clusters equals four times the Al content (at.%). For example, in Cu_75_Ni_20_Al_5_ (100 at.%), x is calculated as 20 at.% (0.2). Thus, the cluster formula is γ-{(Cu, Ni)_16_}_80%_ + γ′-{Al_4_Ni_8_Cu_4_}_20%_. **When the Ni/Al atomic ratio < 2,** excess Al dissolves in the γ phase, and x equals twice the Ni content (at.%). The corresponding cluster formula becomes γ-{(Cu,Al)_16_}_1-x_ + γ′-{Al_4_Ni_8_Cu_4_}_x_.

Recently, Zhang [17] investigated the Ni/Al atomic ratio (2–3) in Cu-Ni-Al alloys (aged at 500 °C) and found that tensile strength decreased by 3.52% and conductivity decreased by 14.1%. This study confirmed that the Ni/Al atomic ratio of 2 defines the γ′ phase formation threshold and the boundary for excess Ni dissolution into the γ phase (the primary conductive phase). Thus, the Ni/Al atomic ratio is further confirmed to be 2 (weight ratio 4.36), which governs γ′ phase formation in high-Cu alloys (Cu > 60 at.%), deviating from the theoretical ratio of 3. The lattice mismatch between the γ phase (Cu) and the γ′ phase (Ni_3_Al) is on the order of 10^−3^ (0.24–0.41%) [25], and the two phases exhibit comparable atomic densities. Therefore, the parameter x, representing cluster unit content, corresponds directly to the γ′ phase volume fraction.

## 3. Construction of Cluster Formula for Cu-Ni-Al Cupronickel Alloys

### 3.1. Elemental Classification in Cu-Ni-Al Cupronickel Alloys

The cluster formula treats phases and alloy compositions as a ternary system, such as Cu-Ni-Al alloys: γ-{(Cu,Ni,Al)_16_}_1−x_ + γ′-{Al_4_Ni_8_Cu_4_}_x_. For Cu-Ni-Al cupronickel alloys, the alloying elements are Mg, Nb, Si, Cr, Mn, and Fe; the partitioning ratio of these alloying elements also determines the cluster formula. According to the solubility behavior of these alloying elements between the γ phase (Cu solid solution) and the γ′ phase (Ni_3_Al solid solution), these elements are classified as follows: γ phase elements (Cu-like elements); γ′ phase elements: the Al-like elements (occupying Al sites); and Ni-like elements (occupying Ni sites). In particular, Fe and Mn are classified as both types of elements simultaneously, and their partitioning ratios need to be determined from the measured composition of the γ′ phase, as described in later sections.

The γ phase elements (Cu-like elements) contain Cu and Ni, both of which exhibit limited solubility in the γ phase (Cu solid solution). When these elements exist in the γ phase, they are regarded as Cu-like elements (note that Cu and Ni also participate in forming the γ′ phase simultaneously). According to the Cu-Mg [26,27] and Ni-Al-Mg [26,27] phase diagrams, the γ′ phase contains only 2 at.% Mg, and Mg predominantly dissolves in the γ phase. Therefore, Mg is a typical Cu-like element.

The γ′ phase elements are classified into two groups: Al-like elements and Ni-like elements. Ochial et al. [28] found that when Cu dissolves in the γ′ phase (Ni-Al-Cu), the γ′ phase region aligns parallel to the Ni-X axis, indicating that Cu replaces Ni in the γ′ phase and acts as a Ni-like element. In contrast, Nb and Si shift the γ′ phase region parallel to the Al-X axis, suggesting they replace Al and act as Al-like elements. Notably, Fe, Mn, and Cr entering the γ′ phase cause its region to expand toward intermediate regions, implying these elements exhibit mixed behavior (acting as both Ni-like and Al-like elements).

For Fe, Mn, and Cr showing mixing behavior, Vowles et al. [29] indicated that the chemical formula of the γ′ phase is (Ni,Cu)_3_(Al,Fe,Mn). X-ray diffraction (XRD) analysis of the extracted phase identified Ni_3_Al, Ni_3_Fe, and Ni_3_Mn, suggesting that Fe and Mn primarily occupy Al sites in the γ′ phase, thereby acting as Al-like elements. However, Nicholls et al. [30] observed that Fe occupies both Al and Ni sites simultaneously, leading to its classification as a dual-substitution element (both Al-like and Ni-like). Similarly, Mn is similarly categorized as both a Cu-like and Al-like element based on the Cu-Mn and Ni-Al-Mn phase diagrams [31], which indicate 33 at.% Mn solubility in the γ phase (Cu) and 20 at.% Mn in the γ′ phase at 500 °C. For Cr, Tuck et al. [16] pointed that small additions of Mn or Cr enhance the γ′ phase ordering degree, with both elements exhibiting analogous alloying effects in Cu-Ni-Al systems. This similarity justifies classifying Cr as an Al-like element within the γ′ phase.

The elemental classification is as follows: γ phase elements (Cu-like): Cu, Ni, Al, Mg, Mn; γ′ phase elements (Al-like): Al, Nb, Si, Cr, Mn, Fe; γ′ phase elements (Ni-like): Ni, Fe, Cu. It is worth noting that behind the site preference of different element atoms, there lies certain physical factors driving the atom replacement. Besides the solid solubility that determines the Cu-like elements, the main physical parameters guiding the atom classification are atomic size and mixing enthalpy. The site selection of alloying elements is influenced by the atomic radii, i.e., elements tend to occupy sites whose atomic radius is closer to their own. Meanwhile, for the mixing enthalpy between elements, certain elements favor occupying the type of site with larger mixing enthalpy, resulting in higher stability of the solid solution. For example, element M with ΔH_Ni-M_ < ΔH_Al-M_, favors occupation of Al sites. By considering comprehensively the factors of solid solubility, atomic size, and mixing enthalpy, a “degree of likeness” with Cu, Al, and Ni can be readily developed.

Applying this classification to the Cu-Ni-Al cupronickel alloys Marinel220, the measured γ′ phase composition is Cu_14.1_Ni_59.3_Mn_2.7_Al_15.4_Fe_2.7_Nb_3.8_Cr_1.0_Si_1.0_ (at.%) [5,9], with the following allocations: Ni-like elements (Ni_59.3_Fe_2.7/2_Cu_14.1_; 74.75 at.%); Al-like elements (Al_15.4_Fe_2.7/2_Nb_3.8_Cr_1.0_Si_1.0_Mn_2.7_; 25.25 at.%); and atomic ratio (Ni-like/Al-like): 74.75/25.25 ≈ 2.96 (close to the theoretical ratio 3). Notably, Fe is distributed in a 1:1 ratio between Ni-like and Al-like roles.

Fe and Mn are classified into both types of elements simultaneously (Fe: Ni-like and Al-like elements; Mn: Cu-like and Al-like elements). Nicholls et al. [30] found that 54%Fe occupies Ni sites, while 9.3 at.% Fe dissolves in the γ′ phase. As Fe content increases, its Ni-site occupancy stabilizes near 50%, supporting a 1:1 partitioning ratio between Ni-like and Al-like roles.

The partitioning ratio of Mn depends on the γ′ phase volume fraction (x), derived from Marinel 220 composition Cu_68.88_Ni_19.60_Mn_5.00_Al_4.00_Fe_1.30_Nb_0.50_Cr_0.50_Si_0.22_ (at.%) and its γ′ phase composition Cu_14.1_Ni_59.3_Mn_2.7_Al_15.4_Fe_2.7_Nb_3.8_Cr_1.0_Si_1.0_ (at.%). Let M represent the partitioning ratio of Mn acting as an Al-like element (Cu-like fraction = 1 − M). Ni-like elements: Ni_19.60_Fe_1.30/2_ (20.25 at.%) and the Al-like elements: Al_4.00_Fe_1.30/2_Nb_0.50_Cr_0.50_Si_0.22_Mn_5M_ (totaling 5.87 + 5 M at.%). Since M < 1, the atomic ratio (Ni-like/Al-like) is 20.25/(5.87 + 5 M) > 2. As previously established, when this ratio exceeds 2, the γ′ phase volume fraction is defined as four times the Al-like content: 4 × (5 M + 5.87). From the measured Mn content in γ′ phase (2.7 at.% [9]) and 5 at.% Mn (in alloy composition), the equation is 4 × (5.87 + 5 M) ×2.7% = 5. Solving this equation yields M ≈ 0.142 (1/7). Thus, Mn partitions as 1/7 Al-like and 6/7 Cu-like.

The above classification of elements into Cu-like, Ni-like, and Al-like types is a crucial strategy for simplifying the complexity of multi-component Cu-Ni-Al cupronickel alloys, which is an effective approach to map their specific compositions onto the Cu-Ni-Al ternary phase diagram (Figure 6a, Page 9), providing a clear visual representation of their distribution.

### 3.2. Cluster Formula for Cu-Ni-Al Cupronickel Alloys

Considering the complex compositions of Cu-Ni-Al cupronickel alloys, the construction of the cluster formula includes the following steps: **(1)** Based on the cluster model, the cluster formula for the theoretical Cu-Ni-Al ternary system was constructed as γ-{(Cu,Ni,Al)_16_}_1−x_ + γ′-{Al_4_Ni_12_}_x_ (as discussed in Section 2.1). **(2)** Considering the dissolvability of Cu in the γ′ phase (discussed in Section 2.2), the cluster formula was further specified for real Cu-Ni-Al ternary alloys as follows: γ-{Cu_16_}_1−x_ + γ′-{Al_4_(Ni,Fe)_8_Cu_4_}x. **(3)** For multi-component industrial Cu-Ni-Al alloys, the classification of alloying elements was first performed. As discussed in Section 3.1, the alloying elements can be classified as follows: Cu-like elements: Cu, Ni, Al, Mn, Mg; Ni-like elements: Ni, Fe,Cu; and Al-like elements: Al, Nb, Si, Cr, Mn, Fe. **(4)** Subsequently, by employing the formula developed for Cu-Ni-Al ternary alloys in step (2), the cluster formula for Cu-Ni-Al cupronickel alloys was finally constructed.

Additionally, if an excess of Nb is added, the γ″-Ni_3_Nb phase [9] will form. To simplify the element classification and the description of the alloy, the cluster formula for Cu-Ni-Al cupronickel alloys was derived as follows: γ-{Cu_16_}_1−x_ + γ′-{Al_4_Ni_12_}x, where x represents the volume fraction of the precipitated phase. The cluster formula of the γ phase is {Cu_16_}_1−x_, where “Cu” represents the Cu-like elements, including Cu, Ni, Al, Mn, and Mg. The cluster formula of γ′ is {Al_4_Ni_12_}x, where “Al” represents the Al-like elements: Al, Nb, Si, Cr, Mn, Fe; and “Ni” represents the Ni-like elements including Ni, Fe and Cu. Noting this, based on the discussion in Section 2.2, the γ′ phase can contain 25 at.% Cu occupying the Ni-site for Cu-based alloys with adequate Cu element. Consequently, the above cluster formula was further specified as γ-{Cu_16_}_1−x_ + γ′-{Al_4_(Ni,Fe)_8_Cu_4_}_x_.

According to the ratio of Al-like (4):Ni-like(Ni, Fe) (8):Ni-like (Cu) (4) atoms being 1:2:1 in the (γ′ + γ″) phase cluster formula: when the atomic ratio (Ni-like/Al-like) < 2, there is an Al-like excess, and the (γ′ + γ″) phase volume fraction is twice the Ni-like elements’ content (Ni + Fe/2; at.%); conversely, if the atomic ratio (Ni-like/Al-like) ≥ 2, there is a Ni-like excess, and the (γ′ + γ″) phase volume fraction is four times the Al-like elements’ content (Al + Nb + Si + Cr + Mn/7 + Fe/2; at.%). In the following section, the specific steps for constructing the cluster formula of Cu-Ni-Al cupronickel alloys are presented, including BAl6-1.5 (Al-like excess) and Marinel 220 (Ni-like excess).

**If the atomic ratio (Ni-like/Al-like) < 2**, there is an Al-like excess, as exemplified by the BAl6-1.5 (91.8Cu-6.0Ni-1.5Al-0.2Mn-0.5Fe). First, the nominal composition (wt.% from Table 1) is converted to Cu_89.45_Ni_6.33_Al_3.44_Mn_0.23_Fe_0.55_ (at.%). According to the elemental classification, Ni-like: Ni_6.33_Fe_0.55/2_ (6.605 at.%) and Al-like: Al_3.44_Mn_0.23/7_Fe_0.55/2_ (3.75 at.%). The atomic ratio (Ni-like/Al-like = 1.76) < 2, meaning that the quantity of Al-like elements is excessive and abundant Al dissolves in the γ phase, and the (γ′ + γ″) phases volume fraction is controlled by the Ni-like elements and is twice their content (13.21 = 2 × 6.605; at.%). The cluster formula is γ-{(Cu,Al,Mn)_16_}_86.79%_ + γ′-{(Al,Mn,Fe)_4_(Ni,Fe)_8_Cu_4_}_13.21%_. In the γ′ phase cluster formula, the ratio of Al-like (1/7Mn + Fe/2):Ni-like (Ni + Fe/2):Cu is 1:2:1. Therefore, Al-like = 6.605 × 1/2 = 3.303 at.%; and Cu = 6.605 × 1/2 = 3.303 at.%. Considering Mn and Fe partitioning, 6/7 of Mn (0.23 at.%) is assigned to Cu-like elements; and 1/7 of Mn (0.23 × 1/7 ≈ 0.033 at.%) and Fe (0.55/2 ≈ 0.275 at.%) are Al-like. The Al content in the γ′ phase is calculated as follows: 2.99 at.% = Al-like (total 3.303) − Mn (in γ′ phase; 0.033; Al sites) − Fe (in γ′ phase; 0.275; Al sites). For the γ phase cluster formula: Al: 0.45 at.% = total Al (3.44) − Al (in γ′ phase; 2.99); Mn: 0.23 × 6/7 ≈ 0.197 at.%; Cu: 86.15 at.% = Cu (total 89.45) − Cu (in γ′ phase, 3.303). Finally, normalizing the element contents in the γ and γ′ phases and multiplying by 16 yields the cluster formula: γ-{Cu_15.88_Al_0.08_Mn_0.04_}_86.79%_ + γ′-{(Al_3.63_Mn_0.04_Fe_0.33_)_4_Ni_7.67_Fe_0.33_Cu_4_}_13.21%_.

**If the atomic ratio (Ni-like/Al-like) ≥ 2**, there is a Ni-like excess, as exemplified by the Marinel 220 (72.30Cu-19.0Ni-1.8Al-4.5Mn-1.2Fe-0.7Nb-0.4Cr-0.1Si), with the atomic composition Cu_68.88_Ni_19.60_Al_4.00_Mn_5.00_Fe_1.30_Nb_0.50_Cr_0.50_Si_0.22_ (at.%). According to the elemental classification: Ni-like (Ni_19.60_Fe_1.30/2_; totaling 20.25 at.%), and Al-like (Al_4.00_Mn_5/7_Fe_1.30/2_Nb_0.50_Cr_0.50_Si_0.22_; totaling 6.58 at.%). The atomic ratio (Ni-like/Al-like = 3.08) ≥ 2, indicating an excess of Ni-like elements and Ni dissolve in the γ phase. Consequently, the (γ′ + γ″) phases volume fraction is controlled by the Al-like elements (6.58) and is four times their content (4 × 6.58 = 26.32). The cluster formula is expressed as γ-{(Cu,Ni,Mn)_16_}_73.68%_ + (γ′ + γ″)-{(Al,Nb,Si,Cr,Mn,Fe)_4_(Ni,Fe)_8_Cu_4_}_26.32%_. In the (γ′ + γ″) phase cluster formula, the ratio of Al-like (1/7Mn + Fe/2):Ni-like (Ni + Fe/2):Cu is 1:2:1. Therefore, Ni-like = 13.16 (2 × 6.58); Cu = 6.58 at.%. Based on the partitioning of Mn and Fe: Ni-like elements include Fe (1.30/2 = 0.65 at.%) and Ni (13.16 − 0.65 = 12.51 at.%); and Al-like elements include Mn (5 × 1/7 ≈ 0.71 at.%), Fe (1.30/2 = 0.65 at.%), Nb (0.50 at.%), Cr (0.50 at.%), and Si (0.22 at.%). For the γ phase cluster formula: Ni = Ni (total 19.60 at.%) − Ni (in (γ′ + γ″)phase; 12.51 at.%) = 7.09 at.%; Mn = Mn (total 5.00 at.%) × 6/7 ≈ 4.29 at.%. Normalizing the element contents in γ and (γ′ + γ″) phases and multiplying by 16 yields the cluster formula: γ-{Cu_13.53_Ni_1.54_Mn_0.93_}_73.68%_ + (γ′ + γ″)-{(Al_2.45_Nb_0.30_Si_0.13_Cr_0.30_Mn_0.43_Fe_0.39_)_4_ Ni_7.61_Fe_0.39_Cu_4_}_26.32%_. As shown in Table 2, the (γ′ + γ″) phase volume fraction of Cu-Ni-Al cupronickel alloys ranges from 13.24% to 26.32%. BAl13-3 (26.28%), C72400 (26.17%), C72420 (24.84%), and Marinel 220 (26.32%).

### 3.3. Young’s Modulus Predicted by Cluster Formula

In the previous section, the phase volume fraction and the relative elemental compositions (in the γ and (γ′ + γ″) phases) were determined. Notably, these values often exhibit a linear correlation with the Young’s modulus of both individual phases and the alloy [32]. This section aims to calculate the Young’s modulus of industrial alloys using the cluster formula (Table 2).

First, the upper theoretical value of the (γ′ + γ″) phase volume fraction was derived as 73.68% γ′ and 26.32% (γ′ + γ″), representing the maximum value achievable by the industrial alloy’s composition. Consequently, the iso-strain Voigt model [33] was applied to calculate the upper theoretical Young’s modulus of multiphase alloys:

E_Alloy_ = E_γ_ × V_γ_ + E_γ′+γ″_ × V_γ′+γ″_, (1)

The Young’s modulus E_γ_ of the γ phase (Cu solid solution) was calculated by considering the relative content of alloying elements (Ni, Al, Mn, and Mg) dissolved in the γ phase derived from the cluster approach. A linear relationship of the weighted contributions for each alloying element was assumed. According to the work of Zhang [34], when different alloying elements were introduced into the Cu solid solution, the variation in their influence on the Young’s modulus was slight. Moreover, in the present study, the content of alloying elements in Cu solid solution was small. Consequently, the linear relationship assumption of the weighted contributions for each alloying element can cause only negligible deviation of the Young’s modulus E_γ_. For example, in Marinel 220, the γ phase composition is Cu_62.30_Ni_7.09_Mn_4.29_ (at.%; Table 2). After normalization (Table 3), the composition becomes Cu_84_Ni_10_Mn_6_. The Young’s modulus of the γ phase was calculated as 123.2 GPa based on weighted contributions (Cu:120 GPa [35]; Ni:207 GPa [35]; Mn:28.9 GPa [35]):

E_γ_ = E_Cu_ × 84% + E_Ni_ × 10%+ E_Mn_ × 6% = 123.2 GPa. (2)

The Young’s modulus calculation is based on the Voigt model, assuming ideal stoichiometry for the γ and γ′ phases. However, according to the proposed elements classification, for multi-compositional Cu-Ni-Al cupronickel alloys, the γ′ phase elements can be classified into Al-like (Al, Nb, Si, Cr, Mn, Fe) and Ni-like elements (Ni, Fe, Cu). The γ′ phase can deviate from specified stoichiometry in real industrial alloys. The modulus of the γ′ phase is composition-sensitive. First-principles studies in the literature consistently suggest that the incorporation of various elements (Cr, Fe, Mn, Si) into the Ni_3_Al structure generally enhances its elastic modulus compared to the binary compound: Cr (on Al sites) increases the modulus from 204.0 to 216.4 GPa [36]; Si (on Al sites): the Ni_3_Si modulus reaches 264.2 GPa [37]; Mn (on Al sites): the Ni_3_Mn modulus is around 200 GPa, comparable to Ni_3_Al [38]; and Fe (on Al sites): the Ni_3_Fe exhibits a modulus of 244.9 GPa [39].

Based on the information above, the introduction of Al-like elements (Cr, Fe, Mn, Si) into the Ni_3_Al structure can cause certain changes in the elastic modulus. Considering the contents of such elements are generally small, such changes in the elastic modulus are very limited. For the Ni-like elements, i.e., Fe and Cu, due to their structural similarity with Ni, only the slight influence on the elastic modulus can be introduced for non-stoichiometric conditions.

Furthermore, recent study [40] revealed that both Ni_3_Nb and Ni_3_Al exhibit stronger constraints under pressure than other L1_2_ phases. Therefore, in the Cu-Ni-Al system, both Ni_3_Al and Ni_3_Nb are key strengthening phases. In the present study, the effects of the non-stoichiometric phases on the elastic modulus of Cu-Ni-Al cupronickel alloys were ignored; only the key strengthening phases Ni_3_Al and Ni_3_Nb were considered during the calculation. Given that the precipitates comprise 92.5% γ′ and 7.5% γ″ (calculated as 4 × 0.5/26.32 = 7.5%; Ni_3_Nb: 265.3 GPa [40]; Ni_3_Al: 218.5 GPa [41]), the Young’s modulus of the (γ′ + γ″) phase is as follows:

E_γ′+γ″_ = (218.5 GPa × 92.5%) + (265.3 GPa × 7.5%) = 202.1 GPa. (3)

Finally, the Young’s modulus of Marinel 220 was determined as follows:

E_Alloy_ = (123.2 GPa × 73.68%) + (219.4 GPa × 26.32%) = 149.2 GPa. (4)

**Figure 7:**
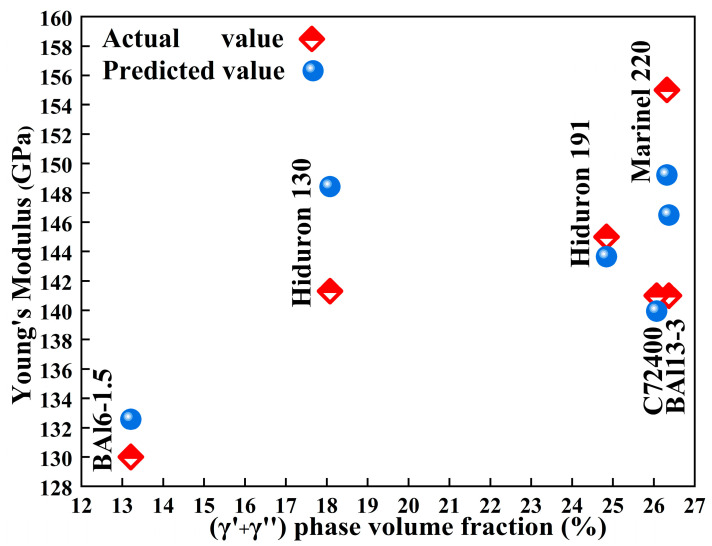
Young’s modulus of the Cu-Ni-Al cupronickel alloys and the predicted values by cluster formula.

The Young’s modulus predicted by the cluster formula for Cu-Ni-Al cupronickel alloys (Table 3 and Figure 7) show the good agreement with the practical alloys, confirming the applicability of the proposed model.

Interfacial tension plays a crucial role in the elastic modulus of composite materials, which is primarily influenced by the lattice misfit between the composed phases of the alloy. Indeed, lattice misfit refers to the degree of geometric mismatch between crystal structures, which determines the interfacial energy. With small lattice misfit, the alloy system tends to maintain a coherent interface while accommodating elastic strain energy. To maintain coherency requires significant energy expenditure due to elastic distortion, leading to a high interfacial tension/energy. For Cu-Ni-Al cupronickel alloys, the lattice misfit between the γ and γ′ phases is relatively large (for example, −0.68 for Marinel220 alloy [5]) due to the introduction of the Nb element. Due to the large lattice misfit, γ′ phases in Cu-Ni-Al cupronickel alloys commonly form spherical shapes [8], leading to low interfacial tension/energy between γ and γ′ phases.

The primary aim of the proposed cluster formula approach is to introduce a new method for classifying elements and characterizing microstructures of Cu-Ni-Al cupronickel alloys, rather than delivering highly precise predictions of Young’s modulus. Therefore, in the present study, the negligible effect caused by the interfacial tension/energy between the γ and γ′ phases were not taken into account. The Voigt model was employed in the elastic modulus calculation, which is, even a relatively simplified approximation, sufficient for the purpose of verifying the model-based approach.

It is worth noting that, for multi-compositional Cu-Ni-Al cupronickel alloys, the chemical element can indeed occupy different structural sites, that is, belong to several elemental groups. Take Mn, for example, which can be classified as both an Al-like and a Cu-like element. To address this issue, the partitioning coefficient was introduced in the present study. The change in the element partitioning coefficient represents the distribution of the element and can affect the volume allocations of the phases of the alloys. For γ′ phase elements in particular, with the changing of partitioning coefficient, the ratio of Ni-like and Al-like elements can change, causing the change in volume fraction of the γ′ phase. (See discussion Section 3.2 on the Ni-like to Al-like elements atomic ratio). The change in the γ′ phase’s volume fraction leads to the change in materials properties. In addition, the elastic modulus of the alloys was partially determined by the composition of each phase, which was also governed by the partitioning coefficient.

## 4. Conclusions

In order to elucidate the correlation between the composition, microstructure, and properties of Cu-Ni-Al cupronickel alloys (characterized by the γ′ phase (Ni_3_Al) and the γ″ phase (Ni_3_Nb) precipitating within the face-centered cubic γ phase matrix), this study established composition formulas based on the cluster plus glue atoms model. Through the classification of alloying elements, the cluster formula of the structural units that carry the composition information was derived. The method was applied to representative Cu-Ni-Al cupronickel alloys, and the main conclusions are as follows:

(1) Alloying elements in Cu-Ni-Al cupronickel alloys were categorized into three groups: Cu-like elements (Cu, Ni, Al, Mg, Mn), Ni-like elements (Ni, Fe, Cu), and Al-like elements (Al, Nb, Si, Cr, Mn, Fe).
(2) The 16-atom cluster formula for Cu-Ni-Al cupronickel alloys was defined as follows:
  γ-{(Cu,Ni,Al,Mn,Mg)_16_}_1−x_ + (γ′ + γ″)-{(Al,Nb,Si,Cr,Mn,Fe)_4_(Ni,Fe)_8_Cu_4_}_x_
(3) According to the 16-atom cluster formula, the precipitates’ volume fraction (x) of Cu-Ni-Al cupronickel alloys is derived as follows: BAl6-1.5 (13.21%), BAl13-3 (26.28%), Hiduron130 (26.17%), C72400 (26.17%), C72420 (24.84%), and Marinel 220 (26.32%).
(4) Young’s modulus, predicted by the cluster formula, exhibits good agreement with the value of Cu-Ni-Al cupronickel alloys, confirming the applicability of the proposed model.

The cluster plus glue atoms model generally applies to typical metals where atomic interactions are weak (to compare, for example, with chemical compounds composed of ionic or covalent chemical bonds). This model is based on Friedel’s oscillation theorem, which describes electron charge screen behavior within the Jellium model, where metal atoms are deprived of their valence electrons and the latter form a so-called valence electron sea. Moreover, this model does not contain any temperature terms, nor any thermal dynamic parameters. Its major advantage lies in identifying the composition unit in a disordered system such as a solid solution or a glass. The composition unit, as well as the associated composition formulas, actually refers to the high-temperature parent state where the structure becomes homogenized and the identification of composition units is made possible, no matter what the real structure becomes in the service condition. Such an endeavor largely avoids the involvements of temperature and other parameters and simplifies the search for optimized chemical compositions. In the present case, the model describes the single-phase high-temperature FCC solid solution state. The solution treatment is exactly conducted over this temperature range. After aging, the γ′ and γ′’ phases are precipitated, and the final microstructure becomes multiphased. However, both the chemical compositions and amounts are rooted in the chemical short-range ordering in the parent FCC solid solution. This is how the alloy composition is formulated and is linked to final microstructure interpretation.

## Figures and Tables

**Table 1:** Composition standard, properties (tensile strength MPa, elongation A%, Young’s modulus GPa), and nominal compositions of the Cu-Ni-Al cupronickel alloys.

		Chemical Compositions (wt.%)								Tensile Strength (A %)	Yield Strength	Young’s Modulus	Original Data
	Grade	Cu	Ni	Al	Mn	Fe	Cr	Mg	Nb				
1	BAl6-1.5, China	bal	5.5–6.5	1.2–1.8	0.2	0.5	___	___	___	638 (7)	___	130	GB5231-2022
2	BAl13-3, China		12.0–15.0	2.3 – 3.0	0.5	1.0	___	___	___	900 (5)	___	141	GB5231-2022
3	UNS C72400-Hiduron130		13.0–16.0	2.3 – 3.0	0.1 – 1.0	0.6 – 1.5	0.5 – max	___	___	850 (10)	630	141.3	ASM Cu-743
4	UNS C72400		11.0–15.0	1.5 – 2.5	1.0 – max	0.1 – max	0.5 – max	0.05–0.40	___	876 (17)	690	141	ASM Book [6,7]
5	UNS C72420- Hiduron191		13.5–16.5	1.0 – 2.0	3.5 – 5.5	0.7 – 1.2	0.5 – max	___	___	725 (18)	430	145	ASM Cu-601
6	UNS C72420- Marinel 220		18.0–25.0	1.6 – 2.2	4.0 – 5.6	0.65 – 0.85	0.36 – 0.48	___	0.55–0.90	870 (15)	700	155	ASM Cu-736

Note: γ phase: Cu solid solution; γ′ phase: Ni_3_Al solid solution; γ′’ phase: Ni_3_Nb solid solution. 1. Nominal composition: 91.80Cu-6.0Ni-1.5Al-0.2Mn-0.5Fe, microstructure: γ and γ′ phases, heat-treatment: 900 °C quenching, 550 °C 2 h aging, air cooling. 2. Nominal composition: 82.35Cu-13.5Ni-2.65Al-0.5Mn-1.0Fe, microstructure: γ and γ′ phases, heat-treatment: 900 °C quenching, 500 °C 2 h aging, air cooling. 3. Nominal composition: 84.70Cu-13.0Ni-2.0Al-0.23Mg, microstructure: γ and γ′ phases, heat-treatment: 900 °C quenching, 500 °C aging, air cooling. 4. Nominal composition: 78.80Cu-14.2Ni-2.3Al-3.9Mn-0.8Fe 14, microstructure: γ and γ′ phases, heat-treatment: ≥600 °C, air cooling. 5. Nominal composition: 81.80Cu-14.4Ni-2.7Al-0.3Mn-0.8Fe 14, microstructure: γ and γ′ phases, heat-treatment: 900 °C solution treatment, ≥500 °C aging. 6. Nominal composition: 72.30Cu-19.0Ni-1.8Al-0.7Nb-4.5Mn-1.2Fe-0.4Cr-0.1Si 14, microstructure: γ, γ′ and γ′’ phases, heat-treatment: 1000 °C solution treatment, air cooling to 600 °C, aging 2 h, air cooling.

**Table 2:** The 16-atom cluster formula of the γ and (γ′ + γ″) phases in Cu-Ni-Al cupronickel alloys.

Elemental classification and its content (Ni-like elements = Ni + Fe/2; Al-like elements = Al + Nb + Si + Cr + Mn/7 + Fe/2)						cluster formula of γ phase			cluster formula of (γ′ + γ″) phase		(γ′ + γ″) volume fraction	
a: Ni-like elements/Al-like elements ≥ 2						{(Cu,Ni,Mn,Mg)_16_}			{(Al,Nb,Si,Cr,Mn,Fe)_4_ (Ni,Fe)_8_Cu_4_}		4 × Al-like	
b: Ni-like elements/Al-like elements < 2						{(Cu,Al,Mn,Mg)_16_}					2 × Ni-like	
	Grades	Cu	Ni	Al	Mn	Fe	Cr	Mg	Nb	Si	at.%	Cluster Formula
1	BAl6-1.5	89.45	6.33	3.44	0.23	0.55					100	
a	γ	86.15	0.00	0.45	0.20						86.79	{Cu_15.88_Al_0.08_Mn_0.04_}
	γ′	3.30	6.33	2.99	0.03	0.55					13.21	{(Al_3.63_Mn_0.04_Fe_0.33_)_4_ Ni_7.67_Fe_0.33_Cu_4_}
2	BAl13-3	78.49	13.93	5.95	0.55	1.08					100	
b	γ	71.92	1.33	0.00	0.47						73.72	{Cu_15.61_Ni_0.29_Mn_0.10_}
	γ′	6.57	12.60	5.95	0.08	1.08					26.28	{(Al_3.62_Mn_0.05_Fe_0.33_)_4_ Ni_7.67_Fe_0.33_Cu_4_}
3	Hiduron 130	77.89	14.85	6.06	0.33	0.87					100	
b	γ	71.35	2.20	0.00	0.28						81.92	{Cu_15.46_Ni_0.48_Mn_0.06_}
	γ′	6.54	12.65	6.06	0.05	0.87					18.08	{(Al_3.70_Mn_0.03_Fe_0.27_)_4_ Ni_7.73_Fe_0.27_Cu_4_}
4	UNS-C72400	81.39	13.51	4.52				0.58			100	
b	γ	76.87	4.47					0.58			73.83	{Cu_15.02_Ni_0.87_Mg_0.11_}
	γ′	4.52	9.04	4.52							26.17	{Al_4_Ni_8_Cu_4_}
5	UNS-C72420	75.03	14.64	5.16	4.30	0.87					100	
b	γ	68.82	2.66	0.00	3.69						75.16	{Cu_14.65_Ni_0.57_Mn_0.78_}
	γ′	6.21	11.98	5.16	0.61	0.87					24.84	{(Al_3.32_Mn_0.40_Fe_0.28_)_4_ Ni_7.72_Fe_0.28_Cu_4_}
6	Marinel 220	68.88	19.60	4.00	5.00	1.30	0.50		0.50	0.22	100	
b	γ	62.30	7.09		4.29						73.68	{Cu_13.53_Ni_1.54_Mn_0.93_}
	γ′ + γ″	6.58	12.51	4.00	0.71	1.30	0.50		0.50	0.22	26.32	{(Al_2.45_Nb_0.30_Si_0.13_Cr_0.30_Mn_0.43_Fe_0.39_)_4_Ni_7.61_Fe_0.39_Cu_4_}

**Table 3:** Young’s modulus (E) of Cu-Ni-Al cupronickel alloys and predicted (E) by cluster formula.

	E_Cu_	E_Ni_	E_Al_	E_Mn_	E_Mg_		Ni_3_Al	Ni_3_Nb			
	120	207	70	28.86	44.8		218.5	265.3			
Grade	Relative Elemental Fraction (at.%)						Eγ	Relative Fraction (at.%)	Eγ′	Predicted E (GPa)	Reported E (GPa)
BAl6-1.5	0.99		0.01			119.5	1		218.5	132.6	130
BAl13-3	0.97	0.02		0.01		120.8	1		218.5	146.5	141
Hiduron130	0.97	0.03				122.6	1		218.5	139.5	141
C72400	0.94	0.05			0.01	123.6	1		218.5	148.4	141.3
C72420	0.91	0.04		0.05		118.9	1		218.5	143.7	145
Marinel 220	0.84	0.10		0.06		123.2	0.925	0.075	222.1	149.2	155

## Data Availability

The original contributions presented in this study are included in the article. Further inquiries can be directed to the corresponding authors.
